
JOURNAL INFORMATION
==============================
NLM Title Abbreviation: Atlanta Med Surg J (1884)
NLM Journal ID: 101251623

Atlanta Medical and Surgical Journal (1884)

Publisher: Biomedical Journal Digitization Project

ARTICLE INFORMATION
==============================
PMCID: PMC9201855
Article version: 1
Subjects: Book Reviews, Pamphlets, Exchanges

Book Reviews

Print publication date: 1899 Feb
Volume: 15
Volume Series: New Ser
Issue: 12
First page: 839
Last page: 844
License: This work is free of known copyright restrictions. For more information, please see PMC Back Issue Digitization.
License URL: https://creativecommons.org/publicdomain/mark/1.0/

==============================

### *BOOK REVIEWS, PAMPHLETS, EXCHANGES.*

### *BOOK REVIEWS.*

[Contributions solicited for review.]

THE PHONENDOSCOPE AND ITS PRACTICAL APPLICATIONS. With 37 illustrations. By Prof. Aurelio Bianchi, its originator, of Parma, Italy. Translated by A. George Baker, A.M., M.D., of Philadelphia. Published by George P. Pilling & Son, of Philadelphia.

This little book, like the one on "Autscopy of the Larynx," is an explanation of and the uses of the Phonendoscope, which means "an instrument which carries to the outside, sounds which originate within an organism." We have not as yet seen the practical workings of this instrument, but we cannot read the author's description without being convinced of its value. The name of Camman should always be held with pride by the American profession, since it was he who first gave us the practical working of binaural stethoscope, and from this Bianchi, by patiently working for fifteen years, has constructed the phonendoscope, which probably bids fair to supersede the old instrument. Its construction is on the idea of the sounds being transmitted through a diaphragm, which thus amplifies and transmits the same just as the diaphragm in the telephone. The instrument is, therefore, much more delicate, and likewise exact, than the old stethoscope; but, like the latter, it takes great experience to become efficient in its use. For instance, the delicacy of the instrument is shown by the fact that in listening to the sounds elicited by percussion, we must simply draw our finger lightly or firmly across the parts, instead of thumping in the old manner. The instrument is so delicate that one is enabled to examine the heart, the lungs, the abdominal viscera, the blood-vessels, the muscular system, and, in fact, nearly all of the organs of the body. For instance, the author says that in fractures of the bones we are enabled to hear crepitus without the slightest difficulty, and that too without subjecting the patient to pain. We are even enabled to hear the contraction of a muscle under the influence of the electric current. Then, again, any number of tubes going to the ear can be attached to the phonendoscope, and thus several can listen at the same time, which would be very valuable for bedside instruction. The author also speaks of "comparative auscultation," meaning the use of two instruments over different areas. For instance, we might listen at the same time to respiration in both lungs, and thus note the differences in the two sides. The author gives numerous photographic illustrations showing the uses of the instrument, and for one who wishes to perfect himself in the use of the phonendoscope, it would certainly be advisable to obtain this book, which is the only authorized translation. The price of the book is only fifty cents.

---

THE PRINCIPLES AND PRACTICE OF MEDICINE. Designed for the Use of Practitioners and Students of Medicine. By William Osler, M.D., Professor of Medicine in the Johns Hopkins University, and Physician-in-Chief to the Johns Hopkins Hospital. Published by D. Appleton & Co., New York.

Six years ago Dr. Osler published the first edition of this work, and now within that space of time this the third is issued from the press, showing the date of July, 1898. This in itself shows the merit of the work. For years in the American medical schools the practice of Flint and Bartholow held sway, then came the excellent work of Loomis, and now this work of Osler seems to have completed a magnificent "triumvirate" of American talent. All of them are works which will remain as classics, and in this the third edition, Dr. Osler has given to the medical profession one of the best works on the practice of medicine which we have. Many of the subjects have been enlarged and not a few entirely rewritten, while distinct additions can be found throughout the book. It would be needless for us to review this work in detail, for such an epitome would even then savor of prolixity, suffice it say that it will always occupy a prominent place as a text-book in all the American medical schools. Since his connection with the Johns Hopkins Hospital, this institution has afforded Dr. Osler an enormous amount of material for scientific and practical study, so that throughout his book one can see the expression of ideas drawn from extensive opportunities for observation. The arrangement of the work is excellent and cannot but commend itself, and in the discussion of each disease a thoroughness and perspicuity is shown which at once fastens itself upon the reader. The publishers have also done their work well.

---

THE SEXUAL INSTINCT. By Dr. James Foster Scott. Published by E. B. Treat & Co., New York.

If "The Sexual Instinct," by Dr. James Foster Scott, had been one of the "classics" found upon the shelves of the libraries of the past generations and read by the men and women of past days, instead of the "Confessions of Rousseau" and the "Decameron," the men, women and children of the present day and generation would have been cleaner, healthier and better morally, mentally and physically. If they had been taught, and had taught their sons, not to say daughters, for daughters have not been taught much of anything in this line, the difference between sexuality and sensuality, we would have had in this day a race of giants; instead of a race composed so very largely of neurasthenics. The book is clean, true, plain spoken, but not too plain, practical and scientific. It ought to be read by physicians, laymen and women. Men and women sin ignorantly; the consequence of such sin is visited upon the third and fourth generation. But since this book is written and they may read, there is no more excuse for such ignorance.

---

ORTHOPEDIC LECTURES. By Dr. A. Sydney Roberts.

This book is a collection of articles by the late A. Sydney Roberts, on club-foot, Pott's disease, knock-knee, bow-legs, spinal orthopedics, flat-foot and chronic articular osteitis of the knee-joint, edited by James K. Young. It was prepared by Dr. Young, the pupil, friend and successor of Dr. Roberts, solely as a tribute to the memory of one whose intellectual attainments, ability as a mechanic and surgeon and sturdy manhood had rooted an affectionate admiration, whose gratitude could best be expressed by arranging a book from some of the writings of Dr. Roberts. The articles are all good. The scheme, the literary references, the evidences of pathological study, the clear outlines for treatment, show that combination of research and application which mark the able man in medicine and surgery.

One lays the book down with the feeling that it has accomplished its purpose. It shows the character of the man, and tells what he was better than could shafts of marble and mounds of earth. HOKE.

---

THE MEDICAL NEWS POCKET FORMULARY. By Dr. E. Q. Thornton, of Philadelphia. Published by Lea Brothers & Co., Philadelphia.

This little pocket formulary is neatly gotten up in leather, and in many respects resembles the appearance of the Medical News Visiting List. As a general thing we are opposed to such books, because it is apt to make a physician, especially if it be a young man, fall into mechanical habits. We believe in every physician studying his cases and making his own prescriptions. This little book, however, is intended more as a *résumé* of the prescriptions which have recently been found by various physicians to be efficacious in certain maladies. For instance, it is impossible for the busy practitioner to read or see reference to every good article which has been written during the year, and yet there may have appeared some remedy of exceptional value which he would desire to use. Dr. Thornton has, in this little pocket formulary, culled all the good remedies from recent literature and put it into this little compact form. As such his book has distinct value.

---

ACROMEGALY. An Essay to which was awarded the Boylston Prize of Harvard University for the year 1898. By Guy Hinsdale, A.M., M.D., of Philadelphia. Published by Wm. M. Warren, Detroit. Price $1.00.

As the explanatory sentence states, this work of 86 pages was a prize essay, first published serially in the monthly medical journal,

*Medicine,* and the author now has issued it in book form, making it therefore a much more handy work of reference. The sub ect has been most thoroughly treated from every standpoint, and such thoroughness is well attested by the long bibliography which we find at the finis. The subject is also well illustrated by photographs of patients who have suffered from acromegaly, and in this way the author has succeeded in placing before the profession not only a very valuable treatise on this subject, but also an exceedingly entertaining one.

---

A Text-Book of Mechano-Therapy (massage and medical gymnastics). Especially prepared for the use of medical students and trained nurses. By Axel V. Grafstrom, B.Sc., M.D., late lieutenant in the Royal Swedish army, late house physician city hospital, Blackwell's Island, New York, with eleven pen-and-ink sketches by the author. Published by W. B. Saunders, 925 Walnut street, Philadelphia. 1899.

This little book will prove to be of considerable aid to the medical student and nurse, in acquiring the rudiments of a very important branch of therapeutics. The author has adopted the system practiced by the Royal Gymnastic Central Institute, Stockholm, Sweden, as a basis for the text, with slight modifications. The pen-and-ink sketches are very appropriate and instructive. We can recommend the work as being a reliable guide to the beginner. S.

---

Transactions of the American Orthopedic Association. Twelfth session. Held in Boston, Mass., May 17, 18 and 19, 1898.

If any one should think that orthopedics does not occupy a prominent place as a distinct specialty, he would very readily discover his mistake after reading these transactions and seeing the presentation of fifty-four such able papers. This volume of transactions is the best we have ever had the pleasure to review, and each contribution shows profound and practical study. Some of the papers are well illustrated, which is always an entertaining feature. This association is certainly to be commended for the good work it is doing.

---

MESSRS. LEA BROTHERS & CO. announce the early publication of *The Practice of Obstetrics. By American Authors.* They announce that it will be a work of exceptional value. Professor Charles Jewett is the editor.

---

### MAGAZINE NOTES.

THE *Scientific American* continues to be filled with good things. Some of its recent illustrations, amplified by means of the text, have been exceedingly interesting and instructive. When placed upon the table in our reception room it is always read with avidity.

THE *Ladies' Home Journal* for January could scarcely be improved upon. There were some delightful character sketches contained in this issue and the illustrations were exceedingly interesting. This is certainly the journal for the family.

---

### REPRINTS RECEIVED.

Coloring Matter and Ferments. By J. F. Peavy, M.D., Asheville, N. C.

Thrombosis of the Lateral Sinus, Dependent upon Suppurative Otitis Media, with Report of Cases.

The Sequelæ of Middle Ear Suppuration, with Report of Cases. Both by Edward B. Dench, M.D., New York.

Diseases of the Ear as a Specialty. By Emil Amberg, M.D., Detroit, Mich.

The Sixth Annual Report of the Sheppard Asylum, Baltimore, Md.
